# Cartilage regeneration and inflammation modulation in knee osteoarthritis following injection of allogeneic adipose-derived mesenchymal stromal cells: a phase II, triple-blinded, placebo controlled, randomized trial

**DOI:** 10.1186/s13287-023-03359-8

**Published:** 2023-06-14

**Authors:** Bahareh Sadri, Mohammad Hassanzadeh, Abolfazl Bagherifard, Javad Mohammadi, Mehdi Alikhani, Kasra Moeinabadi-Bidgoli, Hoda Madani, Dylana Diaz-Solano, Shahedeh Karimi, Mohammad Mehrazmay, Mehdi Mohammadpour, Massoud Vosough

**Affiliations:** 1 Department of Regenerative Medicine, Cell Science Research Center, Royan Institute for Stem Cell Biology and Technology, ACECR, Tehran, Iran; 2 Bone and Joint Reconstruction Research Center, Department of Orthopedics, School of Medicine, Iran University of Medical Sciences, Tehran, Iran; 3 Department of Life Science Engineering, Faculty of New Sciences and Technologies, University of Tehran, Tehran, Iran; 4 Unidad de Terapia Celular - Laboratorio de Patología Celular y Molecular, Instituto Venezolano de Investigaciones Científicas (IVIC), Apartado 21827, 1020-A Caracas, Venezuela; 5 Shahid Beheshti University of Medical Sciences, Tehran, Iran

**Keywords:** Mesenchymal stromal cells, Knee osteoarthritis, Cell-based therapy, Regenerative medicine, Cartilage regeneration, Liquid biomarkers

## Abstract

**Background:**

Intra-articular injection of mesenchymal stromal cells (MSCs) with immunomodulatory features and their paracrine secretion of regenerative factors proposed a noninvasive therapeutic modality for cartilage regeneration in knee osteoarthritis (KOA).

**Methods:**

Total number of 40 patients with KOA enrolled in two groups. Twenty patients received intra-articular injection of 100 × 10^6^ allogeneic adipose-derived mesenchymal stromal cells (AD-MSCs), and 20 patients as control group received placebo (normal saline). Questionnaire-based measurements, certain serum biomarkers, and some cell surface markers were evaluated for 1 year. Magnetic resonance imaging (MRI) before and 1 year after injection was performed to measure possible changes in the articular cartilage.

**Results:**

Forty patients allocated including 4 men (10%) and 36 women (90%) with average age of 56.1 ± 7.2 years in control group and 52.8 ± 7.5 years in AD-MSCs group. Four patients (two patients from AD-MSCs group and two patients from the control group) excluded during the study. Clinical outcome measures showed improvement in AD-MSCs group. Hyaluronic acid and cartilage oligomeric matrix protein levels in blood serum decreased significantly in patients who received AD-MSCs (*P* < 0.05). Although IL-10 level significantly increased after 1 week (*P* < 0.05), the serum level of inflammatory markers dramatically decreased after 3 months (*P* < 0.001). Expressions of CD3, CD4, and CD8 have a decreasing trend during 6-month follow-up (*P* < 0.05), (*P* < 0.001), and (*P* < 0.001), respectively. However, the number of CD25^+^ cells increased remarkably in the treatment group 3 months after intervention (*P* < 0.005). MRI findings showed a slight increase in the thickness of tibial and femoral articular cartilages in AD-MSCs group. The changes were significant in the medial posterior and medial anterior areas of ​​the tibia with *P* < 0.01 and *P* < 0.05, respectively.

**Conclusion:**

Inter-articular injection of AD-MSCs in patients with KOA is safe. Laboratory data, MRI findings, and clinical examination of patients at different time points showed notable articular cartilage regeneration and significant improvement in the treatment group.

*Trial registration*: Iranian registry of clinical trials (IRCT, https://en.irct.ir/trial/46), IRCT20080728001031N23. Registered 24 April 2018.

## Introduction

Knee osteoarthritis (KOA), one of the most common types of osteoarthritis (OA), is mainly characterized by cartilage and subchondral bone impairment. In addition, meniscus and ligament injuries and synovitis could be associated [1]. Furthermore, inflammation is evidenced as a trigger factor in KOA pathogenesis and its association with KOA is directly related to the severity of pain [2]. The rising burden of this chronic degenerative disease peculiarly in developed countries [3] and increased demands for an efficient treatment for KOA have highlighted regenerative medicine as a promising approach. Regenerative medicine represents novel therapeutic modalities for KOA such as platelet-rich plasma (PRP) [4], monoclonal antibodies [5], and cell-based therapies with different cells [6, 7].

Mesenchymal stromal cells (MSCs)-based therapies have been considered as a therapeutic modality for KOA in numerous preclinical [8] and clinical studies [9, 10]. Considering their elite features such as high proliferative capacity, remarkable immunomodulatory features, their notable safe application, the ability to differentiate into chondrocytes, no major ethical concerns, and easy isolation and presence in a wide variety of tissues, MSCs are suitable candidates in regenerative medicine [11]. However, the momentous feature of MSCs is their unique immunomodulatory characteristics [12]. MSCs modulate the production of pro-inflammatory cytokines such as interleukin-6 (IL-6), tumor necrosis factor-α (TNF-α), and interleukin-1β (IL-1β), while usually increase the expression of anti-inflammatory cytokines as interleukin-10 (IL-10) and transforming growth factor β1 (TGF-β1) [13]. This anti-inflammatory capacity has made MSCs a rewarded option for cartilage regeneration and pain relief in KOA [11]. Moreover, lack of co-stimulatory molecules such as CD80, CD86, CD40, and major histocompatibility complex class II (MHC-II), and low expression of major histocompatibility complex class I (MHC-I) on MSCs, results in notable immunomodulatory features and no alloreactivity [13, 14]. These properties of MSCs generate the opportunity for researchers to use allogeneic MSCs instead of the autologous cells in regenerative medicine [15].

Among different sources of MSCs, adipose tissue is the mostly regarded in the last decade. Adipose tissue-derived mesenchymal stromal cells (AD-MSCs) are abundance, accessible, and due to their uncomplicated isolation procedure, and maintaining enhanced differentiation capacity for more extended time they have being applied in many clinical studies [16, 17]. Results of a meta-analysis in 2021, analyzing 19 randomized controlled trials including 811 patients, indicated superiority of AD-MSCs compared to bone marrow-derived mesenchymal stromal cells (BMSCs) [18].

Accustomed monitoring methods for following the effect of MSCs transplantation in KOA are usually limited to imaging-based methods [19, 20]. However, evaluation of some inflammatory or specific biomarkers such as IL-6 and TNF-α in body fluids has been proposed for early diagnosis, and also precise monitoring the effect of MSCs therapy in KOA [10, 21, 22].

In this study, following our previous pilot study which evaluated the clinical and laboratory findings of AD-MSCs transplantation in KOA [23], we present a phase II triple-blinded randomized clinical trial for the evaluation of the efficacy of AD-MSCs for the treatment of KOA.

## Methods

### Study design

The current study represents the phase II of a triple-blinded clinical trial conducted at Shafa Yahyaian hospital, Iran University of Medical Science, between June 2019 and September 2021, and was commensurate with the good clinical practice (GCP) guidelines and the latest version of the Helsinki Declaration. Signed informed consents were obtained from all participants after an accurate explanation of the procedure by the physicians. The trial was monitored and analyzed by the data and safety monitoring board (DSMB; Royan Institute) at Royan Institute. This trial was approved by the Ethics Committee of the Royan Institute (IR.ACECR.ROYAN.REC.1396.138) and registered in the Iranian registry of clinical trials (IRCT, https://en.irct.ir/trial/46) with the following ID code number: identifier IRCT20080728001031N23.

In this study, the stratified block randomization was performed to randomize the patients into AD-MSCs and control groups. Randomization eliminated the possible impact of age and gender on the result of intervention. Permuted block randomization with four blocks/10 patients per block resulted in equal allocation of patients in the treatment and control groups. This randomization provided unbiased selection in treatment assignments. Statistical analysis was conducted to endorse the statistical power for the considered permuted randomization.

After the safety approval which was obtained in the phase I by the assessment and monitoring of three patients [23], phase II was conducted and 40 patients enrolled in two distinct groups, 20 patients at the AD-MSCs group received an intra-articular single dose of 100 × 10^6^ AD-MSCs suspended in 5 ml normal saline (NS), and 20 patients in the control group received 5 ml NS. The patients underwent a single injection of 100 × 10^6^ AD-MSCs in this study; this cell dose has been chosen after the review of some dose escalation studies [24, 25] which declared that high dose (100 × 10^6^) of MSCs transplantation led to more enhancement in clinical and MRI outcomes compared to low or medium dose. In this regard, numerous studies reported the efficacy of this cell dose in KOA without any AEs [26–28]. Although some studies proposed repeated MSCs injection could be better modality in clinic [29, 30], no significant outcome measures have been reported in comparison of single vs. repeated injections. Hence, a single MSCs injection administrated in this study considering the patients’ comfort and also the lack of enough evidence indicating the superiority of repeated MSCs injections in KOA. All the patients, orthopedic surgeon who injected cells, and the interviewer who collected data were blinded to the assigned groups.

**Fig. 1:**
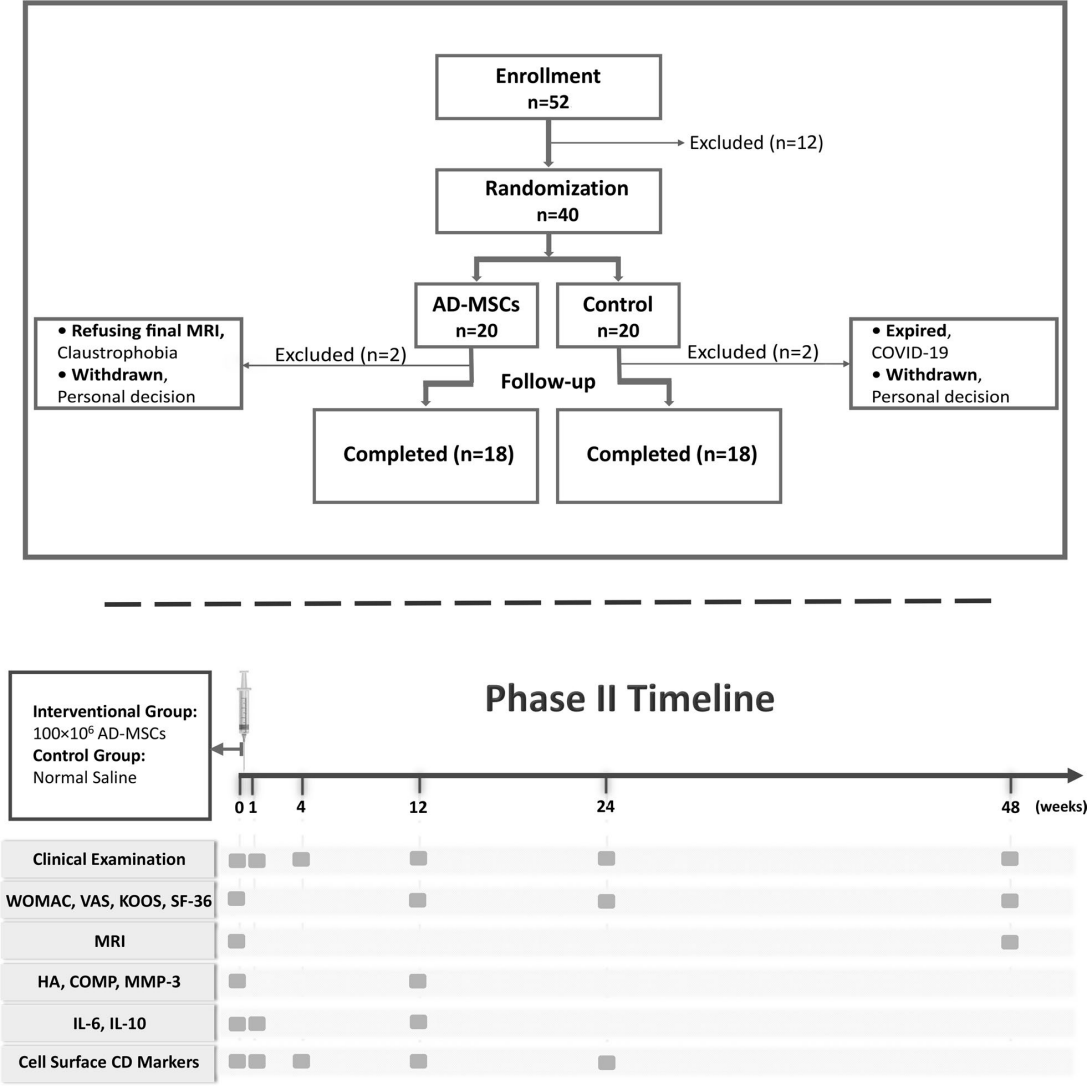
Patients flow diagram and follow-up timeline of the phase II study

The schematic overview of the study design is illustrated in Fig. 1. Eligible participants were aged 18–65 years with mild to moderate symptomatic KOA [Kellgren–Lawrence (K–L) grades of II-III] with visual analogue scale (VAS) equal or more than 3 and knee joint alignment less than 30 degree. These patients were enrolled after signing the informed consent. Details of the inclusion and exclusion criteria are demonstrated in Table 1. The stage of KOA (K-L grades of II-III) in the participants was diagnosed by standard knee X-ray imaging withstanding anterior–posterior (AP) and horizontal lateral radiographic plane.

**Table 1:** Inclusion and exclusion criteria

Inclusion criteria	Exclusion criteria
1. 18 < Age < 65	1. BMI $>$ 35 kg/m^2^
2. Kellgren–Laurence grading: II or III	2. Immune deficiency
3. VAS Index Score $\ge 3$	3. Renal failure (Cr $>$ 2 mg/dL)
4. Knee joint alignment $<$ 30 degrees	4. Hepatitis B, C, HIV, HTLV1-2, CMV infections
5. Informed consent	5. Coagulopathy or using anticoagulant agents
	6. Liver malfunction (ALT or AST $>$ 100 IU/L)
	7. Uncontrolled diabetes (HbA1c > 8.5%)
	8. Heart diseases (EF < 45%)
	9. Allergy to proteins or cultured substances
	10. Pregnancy or lactation
	11. Malignancy
	12. Addiction
	13. Concurrent corticosteroid therapy
	14. Participating in another clinical trial
	15. Unwillingness to cooperate in the study and follow-ups

*VAS* visual analogue scale, *BMI* body mass index, *Cr* creatinine, *ALT* alanine aminotransferase, *AST* aspartate aminotransferase, *HbA1c* hemoglobin A1c, *EF* ejection fraction, *CMV* cytomegalovirus, *HTLV1-2* human T-lymphotropic virus, *HIV* human immunodeficiency virus

Patients in both groups underwent the injection under sterile conditions by an experienced orthopedic surgeon. They were followed up for 12 months and only allowed to take acetaminophen to alleviate the potential upcoming pain during this time. Thirty six out of forty patients completed the 12-month follow-up, while four patients excluded during the study (one patient expired due to COVID19, one patient refused to have final MRI due to claustrophobia, and two patients withdrew from the study based on their personal decision.

### Allogeneic AD-MSCs preparation

Allogeneic AD-MSCs were provided from GMP-certified allogeneic biobank by CellTech Pharmed Co. AD-MSCs were isolated and cultured in IR-FDA-certified clean room facility at CellTech Pharmed Co under PICS/GMP and ISO9001 regulations.

The standard operating procedure for allogeneic AD-MSCs isolation, preparation, and characterization was applied according to GMP regulation at CellTech Pharmed Co. as IR-FDA certified cell production facility same as our previous study [23].

### Outcome measures

Safety assessment, as primary outcome, was conducted by recording any severe adverse events (SAEs) and adverse events (AEs) during the follow-up. AEs and SAEs including allergic reaction, anaphylaxis, fever, chills, pain, injection-site reaction, sudden death, not otherwise specified (NOS), joint swelling, joint stiffness, neurological disorders, skin and subcutaneous tissue disorders; neoplasms benign, malignant, and unspecified gastrointestinal disorders; and infections, renal and urinary disorders were evaluated according to common terminology criteria for adverse events (CTCAE) version 4.0.

Efficacy of AD-MSC’s injection was evaluated and comprised by several questionnaires including Western Ontario and McMaster Universities Osteoarthritis Index (WOMAC), VAS, and knee osteoarthritis outcome score (KOOS), and Short Form 36 (SF-36) questionnaires at the baseline and in 3, 6, and 12 months after injection. Western Ontario and McMaster Universities Osteoarthritis Index (WOMAC), KOOS, VAS, and SF-36 are self-administered questionnaires which completed by patients. The WOMAC consists of 24 items that are divided into three subscales: pain (5 items), stiffness (2 items), and physical function (17 items). Each item was scored on a Likert scale ranging from 0 to 4, with higher scores indicating more severe symptoms. To calculate the total WOMAC score, the raw scores were summed (0–96) and then transformed to a scale of 0–100. Knee osteoarthritis outcome score (KOOS) consists of 42 items, divided into five subscales: pain, symptoms, daily function, sport and recreation, and quality of life. Each item was scored on a Likert scale ranging from 0 to 4, where 0 represents no difficulty or symptom, and 4 represents extreme difficulty or severe symptom. The KOOS score was calculated by summing the scores for each subscale and averaging them, which higher scores indicate better knee function and less severe OA symptoms [31]. The VAS consists of a 10 cm line, with one end representing “no pain” and the other end representing “worst pain imaginable.” Patients were asked to place a mark on the line to indicate their level of pain intensity. The VAS score was calculated by measuring the distance in millimeters between the “no pain” endpoint and the patient’s mark. Higher scores indicated more severe pain intensity. Short Form 36 (SF-36) consists of 36 questions under 8 different subcategories including physical functioning (PF), role physical (RP), body pain (BP), general health (GH), vitality (VT), social functioning (SF), role emotional (RE), mental health (MH) that reflect the physical and mental health status of patients. The score of each subgroup was calculated separately (0–100), and the higher score represented the better patient’s condition [32].

Inflammatory changes following the injection have been assessed by measuring the levels of some specific and inflammatory biomarkers including cartilage oligomeric matrix protein (COMP), hyaluronic acid (HA), matrix metalloproteinase-3 (MMP-3), IL-6, and IL-10 in blood serum at baseline, 1 week, and 3 months after injection. The levels of these biomarkers have been measured by the enzyme-linked immunosorbent assay (ELISA). To measure serum COMP, HA, Il-10, and IL-6 levels using ELISA, human COMP ELISA kit (Cat. No: E1486Hu), human HA Elisa kit (Cat. No: E1374Hu), human IL-10 ELISA kit (Cat. No: E0102Hu), and human IL-6 ELISA kit (Cat. No: DE4640) were used. We followed the manufacturer’s instruction for each kit.

Another monitoring strategy used in this study was evaluation of the cell surface CD markers. Cells obtained from peripheral blood samples were analyzed by flow cytometry for the expression of CD3, CD4, CD8, and CD25 markers. Analysis of cell markers was performed at baseline, and 1, 4, 12, and 24 weeks after injection. In this regard, the samples were processed to obtain a single-cell suspension and remove any debris or aggregates that may interfere with the analysis by adding PBS. The next step was to stain the cells with fluorescent-conjugated antibodies that recognize specific cell surface markers. Different CD markers were detected with their specific antibodies which conjugated with fluorescent dyes to allow their simultaneous detection. After staining, the cells were washed to remove unbound antibodies. Data collection and analysis of the fluorescent intensities were made using a flow cytometer (Becton Dickinson, New Jersey, USA) at Royan institute.

Magnetic resonance imaging (MRI) assessment was conducted at baseline and 12 months after the injection. Fast spoiled gradient-echo (FSPGR) MRI protocol was used in sagittal plane using 3 Tesla system (Magnetom Tim Trio; Siemens, Erlangen, Germany). The thickness of distal femoral cartilage and proximal tibial cartilage was evaluated in femur lateral central (FLC), femur lateral posterior (FLP), femur lateral anterior (FLA), femur medial central (FMC), femur medial posterior (FMP), femur medial anterior (FMA), tibia lateral anterior (TLA), tibia lateral central (TLC), tibia lateral posterior (TLP), tibia medial anterior (TMA), tibia medial central (TMC), and tibia medial posterior (TMP). The one-way repeated measures analysis of variance (ANOVA) has been used to evaluate the validity of cartilage thickness changes at baseline and 1-year post-injection follow-up.

### Statistical analysis

The primary outcome was the difference in WOMAC score between baseline and 12 months. In this regard, the sample size was calculated according to the results of the WOMAC score in a previous study [33]. The *α* risk of 0.05, power of 80%, mean difference in WOMAC score of 0.5, standard deviation of 0.5 were considered in this study which resulted in the sample size of 16 participants per group. We increased the sample size per each group to 20 patients in order to enhance the reliability of our study.

Statistical analysis was conducted using SPSS version 22. Independent sample t test was used for quantitative normally distributed data and chi-square test for qualitative variables. Comparison between groups at different time points were evaluated by one-way repeated ANOVA, and Mauchly’s test of sphericity conducted for testing the assumption of sphericity to validate one-way repeated ANOVA. Furthermore, the reliability of MRI measurements was tested using the intraclass correlation coefficient (ICC) for the two observations. In order to be deemed statistically significant, the *P* value needed to be less than 0.05 in this study.

## Results

### Demographic data

The characteristics of the patients are summarized in Table 2. To negate the effect of the quantitative variables including age, body mass index (BMI) and alignment, and the qualitative variables including gender, K–L grade, and the affected-side knee (left or right), these variables were analyzed in both groups using t test and chi-square tests, respectively. The results are presented in Table 2 and indicated that the effect of the mentioned variables was not significant on the results of this study.

**Table 2:** Demographic characteristics

Variables	AD-MSCs	Control	*P* value	Test
Age (year)	52.85 ± 7.25	56.1 ± 7.21	1	*t* test
BMI (kg/m^2^)	28.37 ± 3.26	29.12 ± 4	0.756	*t* test
Alignment (degree)	3.80 ± 3.34	5.05 ± 2.45	0.06	*t* test
Gender				
F	18	18	1	
M	2	2		Chi-square tests
K–L grade				
II	11	10	1	
III	9	10		Chi-square tests
Side (R/L)				
R	9	12	0.527	
L	11	8		Chi-square tests

*AD-MSCs* adipose-derived mesenchymal stromal cells, *BMI* body mass index, *F* female, *M* male, *K–L grade* Kellgren–Lawrence grade, *R* right knee, *L* left knee

### Laboratory assessments for safety

Table 3 illustrates AEs and SAEs recorded during the study. All participants were followed up for 12 months after intra-articular injection. The AEs reported previously in the phase I of this trial [23] were also observed in 2 patients of the AD-MSCs group in this study. During the first week after injection, both patients had mild and self-limiting local swelling and pain in the injected knee joint, which relieved after 2–3 days. The results of C-reactive protein (CRP), erythrocyte sedimentation rate (ESR), complete blood count (CBC), and different laboratory tests did not show any abnormal parameters related to infection or inflammation. No SAEs were observed in any of the patients.

**Table 3:** Adverse and serious adverse events

Side effects	AD-MSCs	Control	Symptom	Outcome
AEs	2 Patients	–	Swelling of injection-site joint	Self-limited
SAEs	–	–	–	–

*AD-MSCs* adipose-derived mesenchymal stromal cells, *AEs* adverse events, *SAEs* serious adverse events

### Clinical outcomes

VAS, WOMAC, KOOS, and SF-36 questionnaires were used to evaluate the effectiveness of AD-MSCs injection compared to the control group, during the trial (at baseline, 3, 6, and 12 months after injection). Chronological changes of these clinical outcomes in the study groups over the 1-year follow-up are indicated in Table 4.

**Table 4:** Longitudinal change of MRI and clinical outcomes for case and control groups over the 1-year follow-up

Measurement	Group	Baseline	3 months	6 months	12 months	*P* value
WOMAC	AD-MSCs	58.35 ± 13.25	25.90 ± 14.57	16.75 ± 13.81	19.05 ± 14.12	< 0.001
	Control	65.42 ± 14.63	54.73 ± 17.07	55.63 ± 23.17	63.47 ± 20.68	
VAS	AD-MSCs	7.40 ± 1.35	3.75 ± 1.58	3.15 ± 1.87	3.25 ± 1.58	< 0.001
	Control	7.73 ± 1.14	6.10 ± 1.48	7 ± 1.85	7.47 ± 1.54	
*KOOS*						
Pain	AD-MSCs	35.40 ± 15.30	69.80 ± 12.52	79.45 ± 11.97	73.25 ± 16.11	< 0.001
	Control	25.10 ± 14.12	38.78 ± 16.12	32.10 ± 21.02	27 ± 20.72	
Symptom	AD-MSCs	50.45 ± 23.65	79.95 ± 20.52	89.15 ± 15.16	84.35 ± 15.76	< 0.001
	Control	42.15 ± 23.53	57.89 ± 20.40	53.05 ± 20.09	41.68 ± 20.49	
Quality of life	AD-MSCs	16.90 ± 16.94	56.90 ± 18.31	64.60 ± 17.58	60.35 ± 18.94	< 0.001
	Control	13.26 ± 11.47	27.05 ± 15.34	18.78 ± 18.24	16.78 ± 18.33	
Daily function	AD-MSCs	32.80 ± 15.26	68.70 ± 15.16	80.85 ± 13.64	78.10 ± 15.80	< 0.001
	Control	30.52 ± 14.43	42.15 ± 17.87	39.78 ± 22.61	32.89 ± 20.80	
Sport & recreation	AD-MSCs	3.46 ± 16.18	28 ± 20.35	33.50 ± 23.95	21.75 ± 18.58	< 0.001
	Control	6.75 ± 22.37	5 ± 6.45	1.84 ± 5.05	1.05 ± 3.15	
Total KOOS	AD-MSCs	28.30 ± 14.90	60.60 ± 10.20	69.15 ± 12.22	63.75 ± 15.40	< 0.001
	Control	22.05 ± 9.57	34.68 ± 13.50	29.57 ± 16.28	23.84 ± 15.09	
*SF-36*						
PF	AD-MSCs	29.50 ± 11.90	61.75 ± 17.03	72.75 ± 16.66	72.50 ± 17.65	< 0.001
	Control	29.47 ± 11.04	38.42 ± 17.79	32.10 ± 17.58	28.68 ± 14.79	
RP	AD-MSCs	25 ± 18.13	85 ± 27.38	88.75 ± 17.15	96.25 ± 12.23	< 0.001
	Control	26.31 ± 21.20	53.94 ± 31.47	36.84 ± 25.50	31.57 ± 32.10	
BP	AD-MSCs	15.12 ± 15.68	58.87 ± 20	66.50 ± 20.98	65.25 ± 15.89	< 0.001
	Control	15.13 ± 14.58	36.31 ± 22.25	21.44 ± 17.85	23.02 ± 24.44	
GH	AD-MSCs	37 ± 13.70	70.25 ± 17.73	72.50 ± 14.18	71.75 ± 18.01	< 0.001
	Control	31.84 ± 12.15	47.89 ± 17.89	32.05 ± 19.69	26.57 ± 19.72	
VT	AD-MSCs	56.25 ± 12.01	68 ± 15.42	69.75 ± 11.52	71 ± 11.42	< 0.001
	Control	46.31 ± 15.44	58.94 ± 13.49	48.68 ± 14.79	46.05 ± 20.58	
SF	AD-MSCs	30 ± 24.12	64.37 ± 17.33	72 ± 21.36	69.37 ± 16.46	< 0.001
	Control	32.23 ± 22.17	51.57 ± 23.69	37.50 ± 20.83	24.34 ± 23	
RE	AD-MSCs	9.99 ± 19.02	81.66 ± 29.57	86.66 ± 25.13	95 ± 16.31	< 0.001
	Control	14.02 ± 20.22	54.37 ± 37.02	24.55 ± 34.85	19.30 ± 35.69	
MH	AD-MSCs	22.50 ± 19.70	70 ± 15.38	81.25 ± 19.65	85 ± 17.01	< 0.001
	Control	15.78 ± 19.02	48.68 ± 26.96	36.84 ± 26.83	25 ± 26.35	
*MRI (mm)*						
TMA	AD-MSCs	1.86 ± 0.53	–	–	1.98 ± 0.56	< 0.05
	Control	1.49 ± 0.88	–	–	1.37 ± 0.81	
TMC	AD-MSCs	1.78 ± 0.69	–	–	1.79 ± 0.69	0.840
	Control	1.76 ± 0.7	–	–	1.71 ± 0.68	
TMP	AD-MSCs	2.01 ± 0.29	–	–	2.07 ± 0.26	< 0.01
	Control	1.81 ± 0.25	–	–	1.78 ± 0.18	
TLA	AD-MSCs	1.70 ± 0.75	–	–	1.67 ± 0.71	0.997
	Control	1.70 ± 0.66	–	–	1.67 ± 0.65	
TLC	AD-MSCs	2.01 ± 0.42	–	–	2.05 ± 0.41	0.420
	Control	1.90 ± 0.61	–	–	1.88 ± 0.58	
TLP	AD-MSCs	1.77 ± 0.31	–	–	1.82 ± 0.26	0.414
	Control	1.98 ± 0.52	–	–	1.82 ± 0.46	
FMA	AD-MSCs	2.20 ± 0.67	–	–	2.22 ± 0.63	0.397
	Control	1.99 ± 1.01	–	–	1.94 ± 0.98	
FMC	AD-MSCs	1.78 ± 0.82	–	–	1.81 ± 0.83	0.462
	Control	1.59 ± 0.9	–	–	1.57 ± 0.87	
FMP	AD-MSCs	2.36 ± 0.79	–	–	2.39 ± 0.76	0.823
	Control	2.43 ± 0.72	–	–	2.43 ± 0.73	
FLA	AD-MSCs	1.97 ± 0.65	–	–	2.15 ± 0.67	0.677
	Control	2.19 ± 0.45	–	–	2.09 ± 0.48	
FLC	AD-MSCs	1.81 ± 0.43	–	–	1.80 ± 0.39	0.731
	Control	1.86 ± 0.43	–	–	1.84 ± 0.42	
FLP	AD-MSCs	2.20 ± 0.61	–	–	2.23 ± 0.72	0.611
	Control	2.38 ± 0.63	–	–	2.28 ± 0.60	

Values are given as mean ± standard deviations of the mean

*AD-MSCs* Adipose-derived mesenchymal stromal cells, *SF-36* 36-Item Short Form Survey, *PF* physical functioning, *RP* role physical, *BP* body pain, *GH* general health, *VT* vitality, *SF* social functioning, *RE* role emotional, *MH* mental health, *KOOS* Knee injury and Osteoarthritis Outcome Score, *WOMAC* Western Ontario and McMaster Universities Osteoarthritis index, *VAS* visual analog scale, *MRI* magnetic resonance imaging, *FLA* femur lateral anterior, *FMC* femur medial central, *FMP* femur medial posterior, *FMA* femur medial anterior, *TLA* tibia lateral anterior, *TLC* tibia lateral central, *TLP* tibia lateral posterior, *TMA* tibia medial anterior, *TMC* tibia medial central, *TMP* tibia medial posterior

**Fig. 2:**
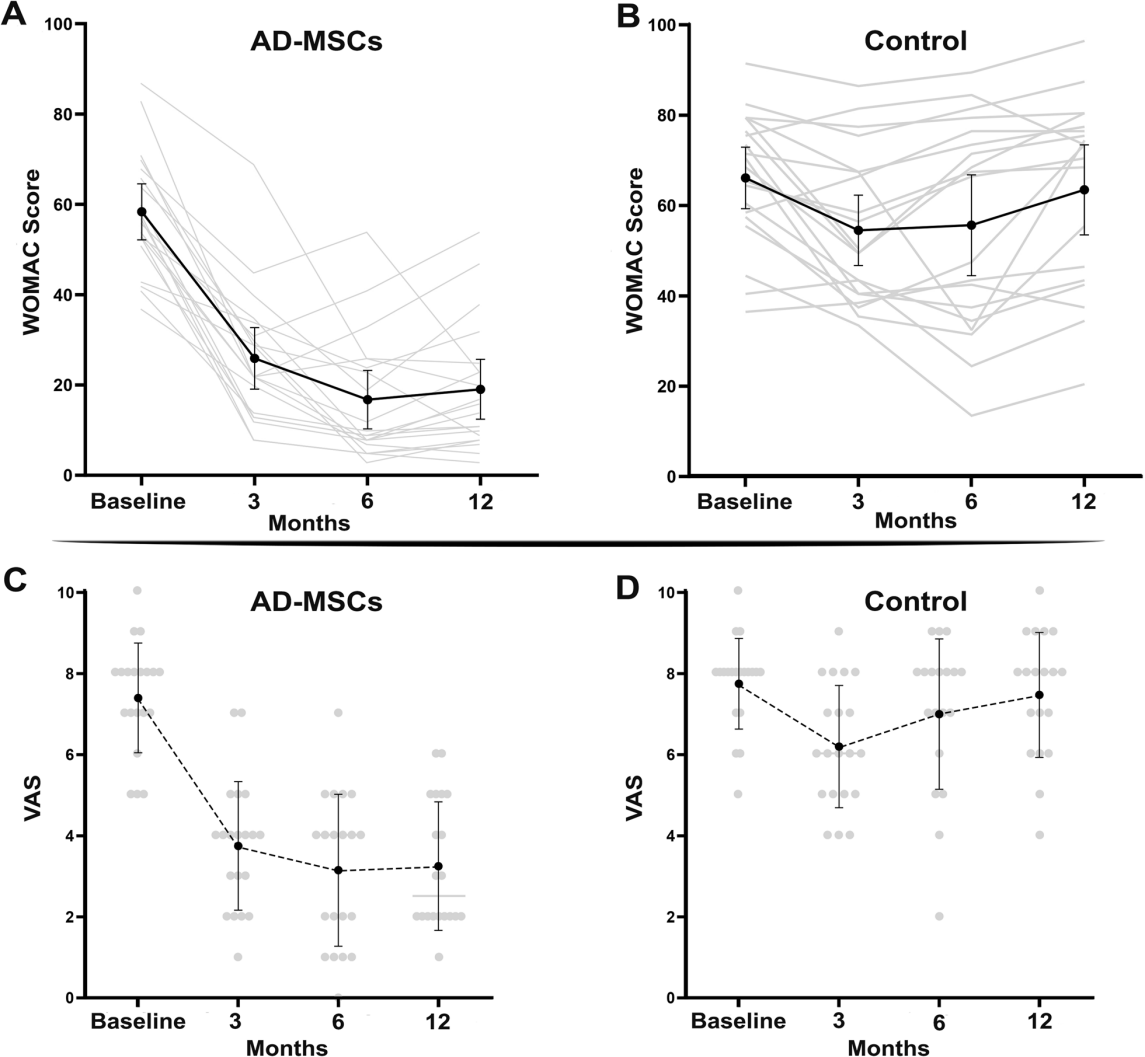
Comparison of the WOMAC (*P* < 0.001) between **A** AD-MSCs and **B** control groups and VAS (*P* < 0.001) between **C** AD-MSCs and **D** control groups during the 12-month follow-up after injection. *WOMAC* Western Ontario and McMaster Universities Osteoarthritis index, *VAS* visual analog scale, *AD-MSCs* adipose-derived mesenchymal stromal cells. Data markers represent means; error bars, 95% confidence interval; and statistical analysis conducted by the one-way repeated measures analysis of variance (ANOVA)

WOMAC questionnaire, the most common questionnaire related to KOA, was used to evaluate the patients’ conditions. Comparison of the results showed that the trend of total WOMAC score in the AD-MSCs group, during the 12-month follow-up, had a significant decrease compared to the control group (*p* < 0.001). The average score of this questionnaire in AD-MSCs group after 6 months of the injection has declined by more than 70% (Fig. 2A, B).

The linear graph in Fig. 2C illustrates the VAS values at baseline, 3, 6, and 12 months after injection in AD-MSCs group. The pain reported by the patients in treatment group in comparison with the control group (Fig. 2D) had a significant decreasing trend (*P* < 0.001).

**Fig. 3:**
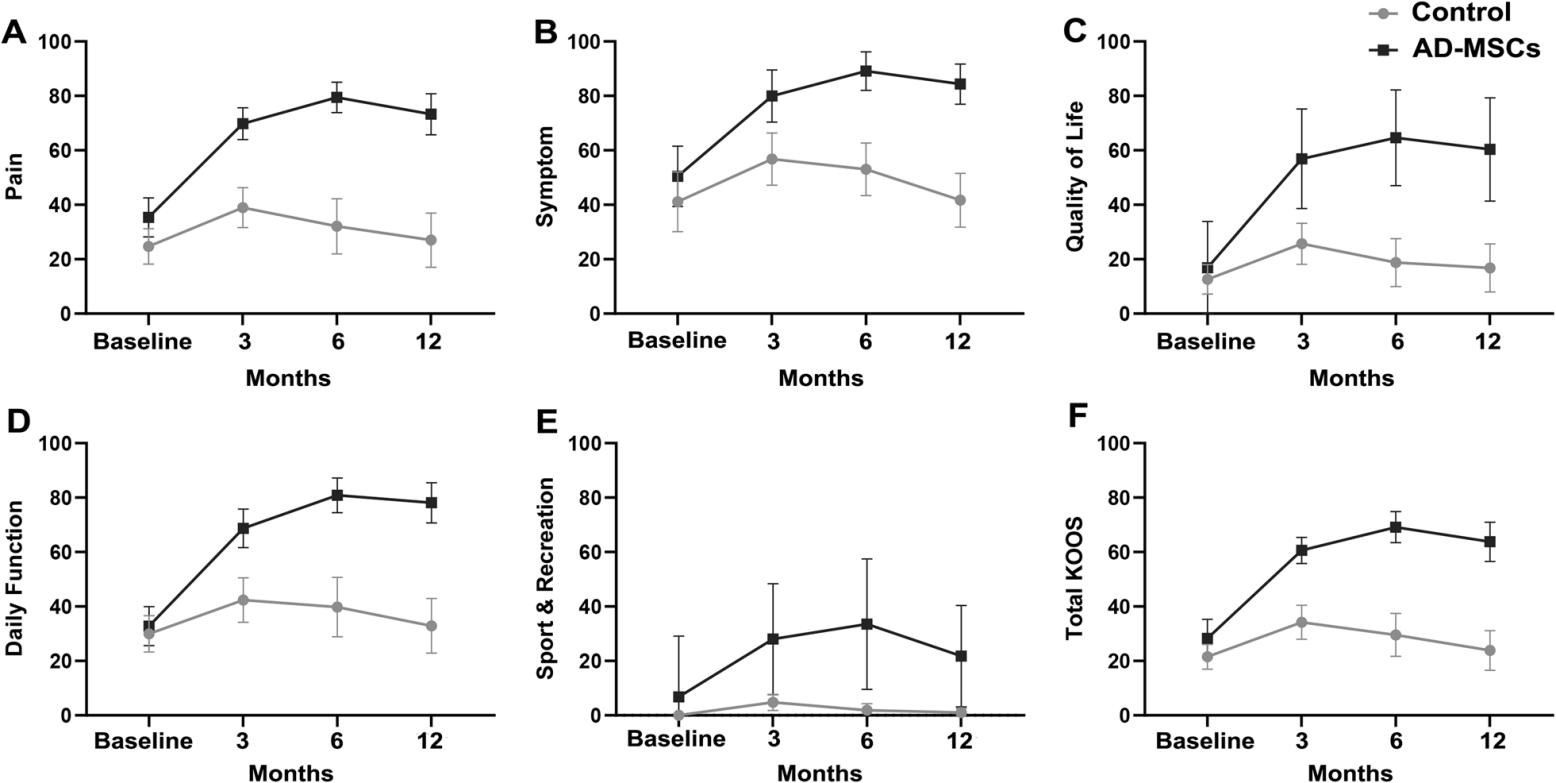
Comparison of the KOOS between AD-MSCs and control groups during 12-month follow-up. **A** KOOS, pain (*P* < 0.001); **B** KOOS, symptom (*P* < 0.001); **C** KOOS, daily function (*P* < 0.001); **D** KOOS, sport and recreation (*P* < 0.001); **E** KOOS, quality of life (*P* < 0.001); and **F** total KOOS (*P* < 0.001). *KOOS* Knee injury and Osteoarthritis Outcome Score, *AD-MSCs* adipose-derived mesenchymal stromal cells. Data markers represent means; error bars, 95% confidence interval; and statistical analysis conducted by the one-way repeated measures analysis of variance (ANOVA)

Figure 3 demonstrates various subgroups and total KOOS and has made a comparison between AD-MSCs and control groups. The results revealed that the total KOOS (Fig. 3F) and also all subclasses including pain (Fig. 3A), symptoms (Fig. 2B), quality of life (Fig. 3C), daily function (Fig. 3D), and sport and recreation (Fig. 3E) increased significantly (*P* < 0.001) in patients who received AD-MSCs. According to Fig. 3, the amounts of KOOS (total and subgroups) in the AD-MSCs group have reached to the highest point 6 months after the injection, while showed a slight decrease at the final time point (12 months) (*P* < 0.001).

**Fig. 4:**
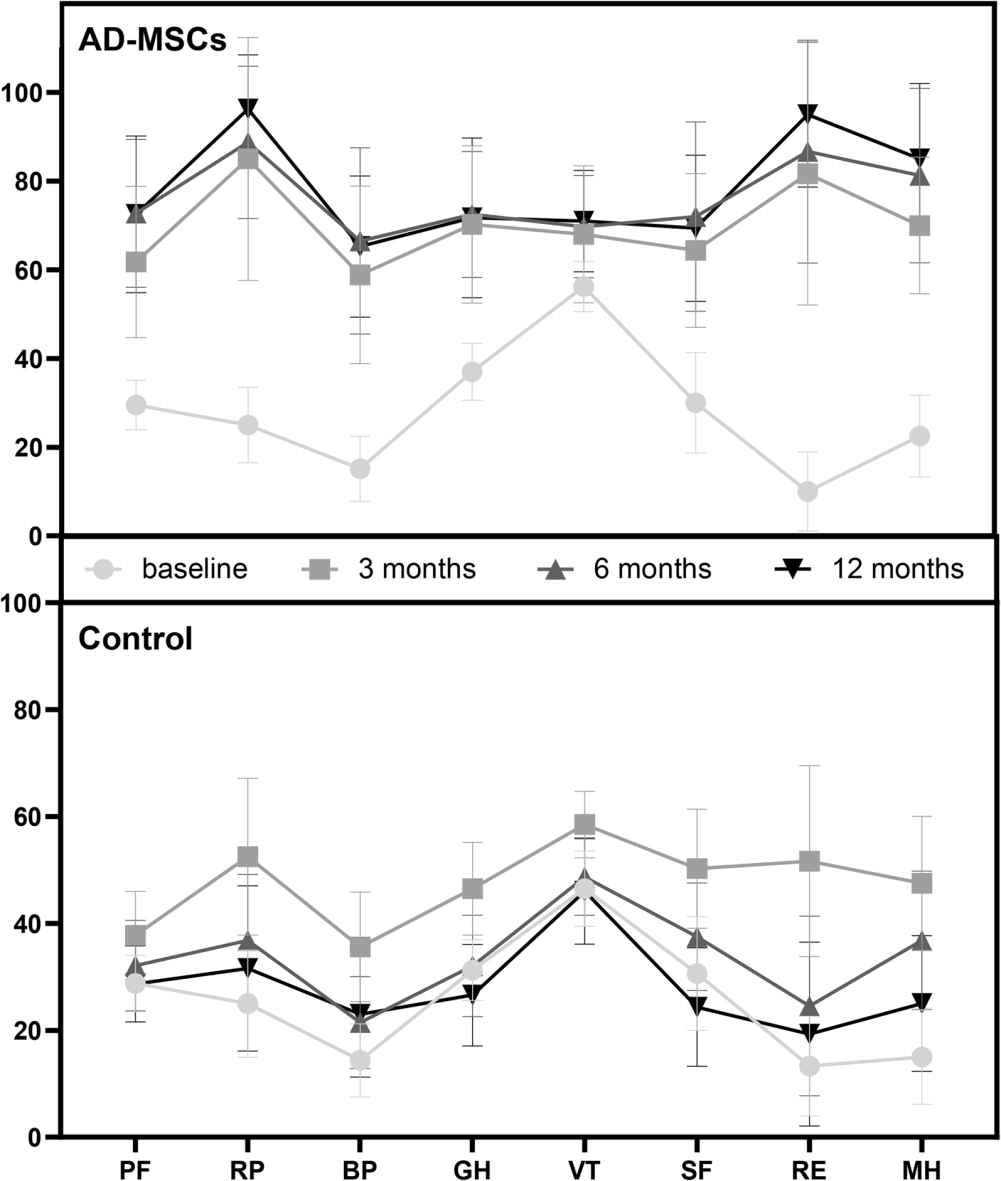
SF-36 outcomes in AD-MSCs and control groups during the 12-month follow-up. *PF* physical functioning (*P* < 0.001), *RP* role physical (*P* < 0.001), *BP* body pain (*P* < 0.001), *GH* general health (*P* < 0.001), *VT* vitality (*P* < 0.001), *SF* social functioning (*P* < 0.001), *RE* role emotional (*P* < 0.001), *MH* mental health (*P* < 0.001). *SF-36* 36-Item Short Form Survey, *AD-MSCs* adipose-derived mesenchymal stromal cells. Data markers represent means; error bars, 95% confidence interval; and statistical analysis conducted by the one-way repeated measures analysis of variance (ANOVA)

Figure 4 shows the results of subgroups of SF-36 questionnaire at different time points in both groups. In general, in the AD-MSCs group, the health condition of the patients improved significantly. This improvement in all subgroups was achieved in 1-year follow-up (*P* < 0.001).

### MRI findings

**Fig. 5:**
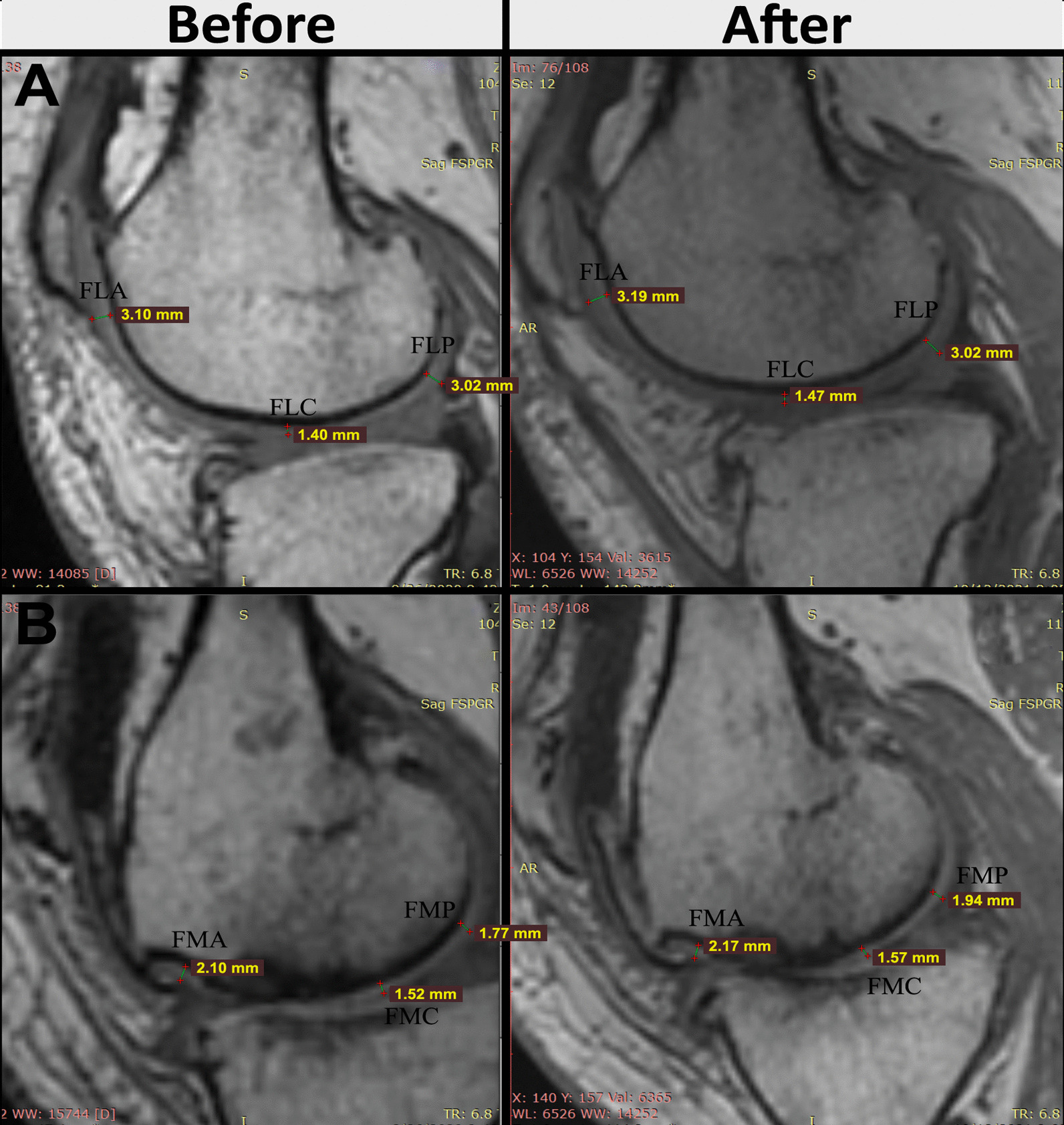
MRI analysis of femural condyle cartilage before and 48 weeks after AD-MSCs injection. **A** lateral radiograph indicates enhancement in anterior, posterior, and central area of femoral condyle cartilage. **B** Medial radiograph revealed increase in the thickness of femoral condyle cartilage in anterior, posterior, and central area. *MRI* magnetic resonance imaging, *FLC* Femur lateral central, *FLP* femur lateral posterior, *FLA* femur lateral anterior, *FMC* femur medial central, *FMP* femur medial posterior, *FMA* femur medial anterior, *AD-MSCs* adipose-derived mesenchymal stromal cells

**Fig. 6:**
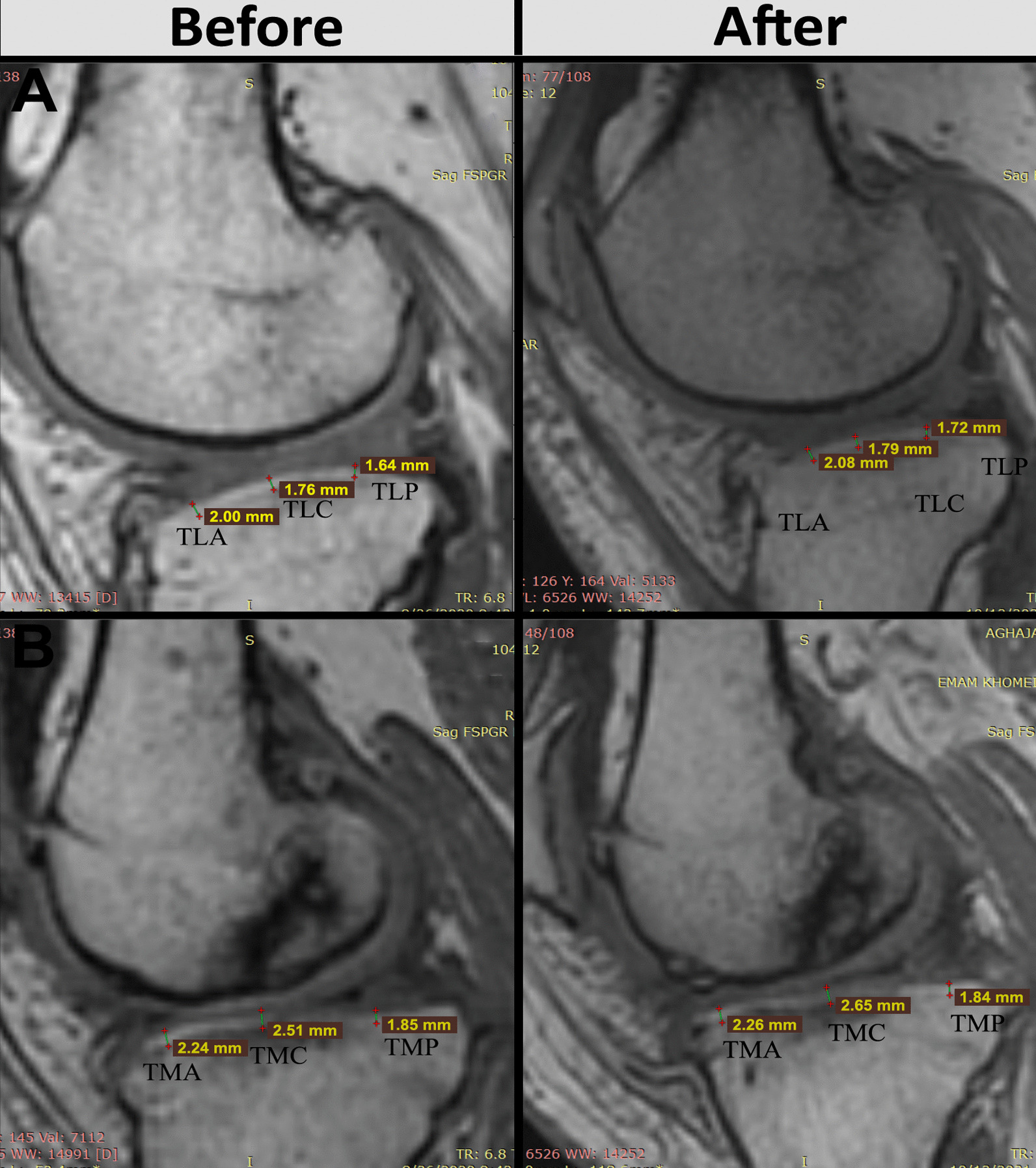
MRI analysis of tibial condyle cartilage before and 48 weeks after AD-MSCs injection. **A** Lateral radiograph indicates enhancement in anterior, posterior, and central area of tibial condyle cartilage. **B** Medial radiograph revealed increase in the thickness of tibial condyle cartilage in anterior, posterior, and central area. *MRI* magnetic resonance imaging, *TLA* tibia lateral anterior, *TLC* tibia lateral central, *TLP* tibia lateral posterior, *TMA* tibia medial anterior, *TMC* tibia medial central, *TMP* tibia medial posterior, *AD-MSCs* adipose-derived mesenchymal stromal cells

Efficacy of AD-MSCs for the treatment of KOA was evaluated by MRI before and 1 year after injection. Figure 5 shows a representative image of the femoral condyle cartilage, in one patient, before and 1 year after AD-MSCs injection. The baseline thickness values for FLA, FLC, FLP, FMA, FMC, and FMP were 3.10 mm, 1.40 mm, 3.02 mm, 2.10 mm, 1.52 mm, and 1.77 mm, respectively, and 1 year after injection increased to 3.19 mm, 1.47 mm, 3.02 mm, 2.17 mm, 1.57 mm, and 1.94 mm, respectively. Figure 6 shows a representative image of the tibial condyle cartilage, in one patient, before and 1 year after injection of AD-MSCs. According to Fig. 6, the thickness before injection for TLA, TLC, TLP, TMA, TMC, and TMP was 2 mm, 1.76 mm, 1.64 mm, 2.24 mm, 2.51 mm, and 1.85 mm, respectively, which reached to 2.08 mm, 1.79 mm, 1.72 mm, 2.26 mm, 2.65 mm, and 1.84 mm 1 year after injection.

**Fig. 7:**
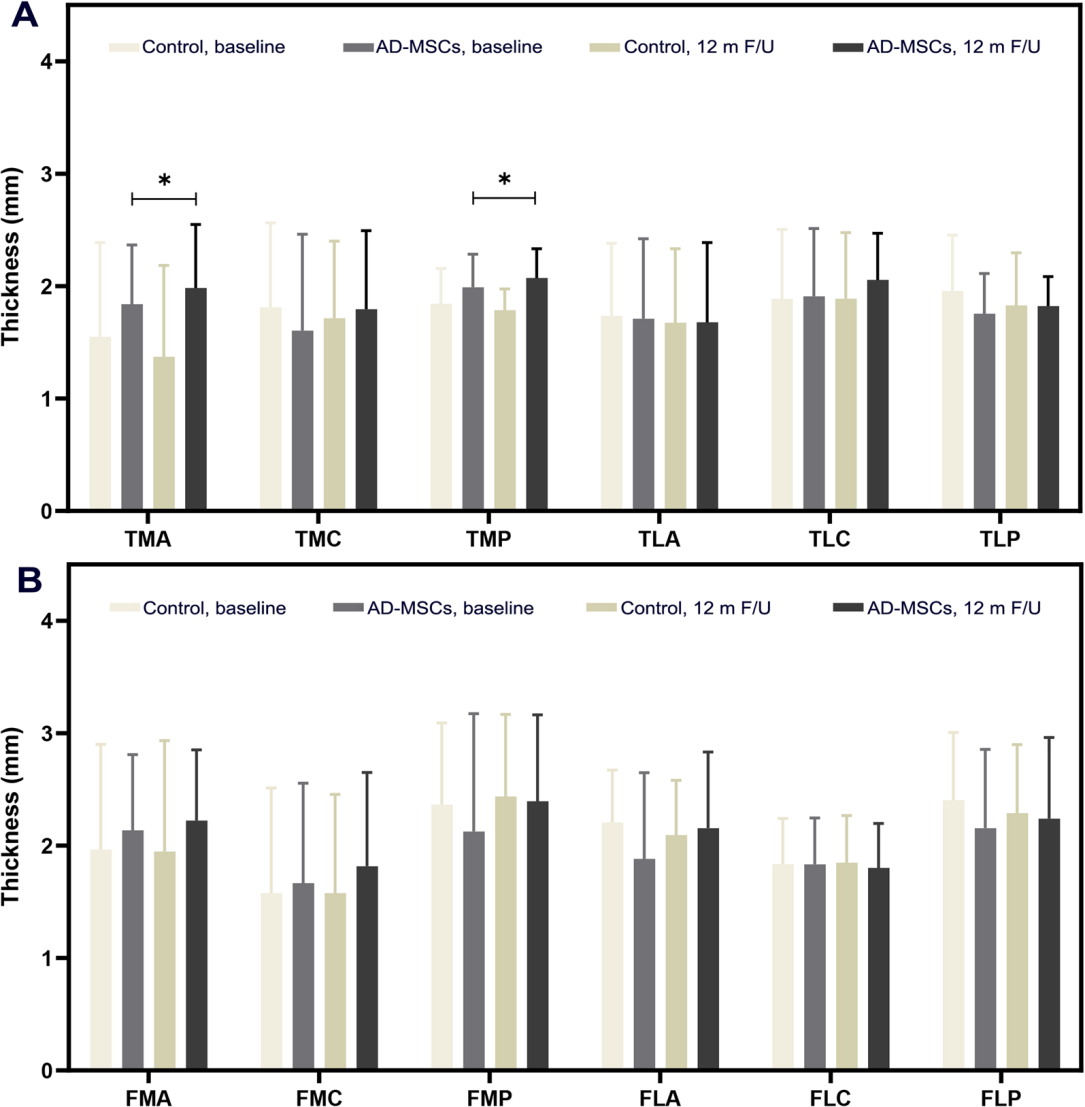
MRI findings. **A** Tibial condyle cartilage thickness. **B** Femoral condyle cartilage thickness. Data markers represent means; error bars, 95% confidence interval; *P < 0.05, between and within groups; and one-way repeated measures analysis of variance (ANOVA). *MRI* magnetic resonance imaging, *FLC* femur lateral central, *FLP* femur lateral posterior, *FLA* femur lateral anterior, *FMC* femur medial central, *FMP* femur medial posterior, *FMA* femur medial anterior, *TLA* tibia lateral anterior, *TLC* tibia lateral central, *TLP* tibia lateral posterior, *TMA* tibia medial anterior, *TMC* tibia medial central, *TMP* tibia medial posterior, *AD-MSCs* adipose-derived mesenchymal stromal cells, *m* months, *F/U* follow-up

Thickness changes in tibial and femoral condyle cartilages are analyzed in Fig. 7. Cartilage thickness in 12 different points of the proximal tibia and distal femur has been assessed. The comparison between MRI findings at the baseline and 1 year post-injection in AD-MSCs and control groups revealed increased thickness in TLC, TLP, TMA, TMC, TMP, FLP, FLA, FMC, FMP, and FMA, in the AD-MSCs group. However, the improvement was just significant in TMA and TMP with *P* < 0.05 and *P* < 0.01, respectively. The ICC was 0.91 for two observations indicating the excellent reliability, and the means thickness in each point is illustrated in Fig. 4. Table 4 indicates the cartilage thickness alterations in details.

### Biomarkers assessment in blood samples

**Fig. 8:**
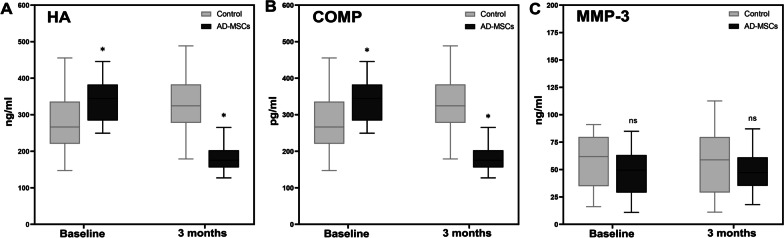
Serum level of biomarkers. **A** HA levels decreased significantly in AD-MSCs group after 3 months (*P* < 0.05). **B** Levels of COMP declined remarkably in AD-MSCs group after 3 months (*P* < 0.05). **C** Level of MMP-3 does not change remarkably in AD-MSCs in comparison with the control group. Data markers represent means; error bars, 95% confidence interval; *P < 0.05, ns: not significant, between and within groups; and one-way repeated measures analysis of variance (ANOVA)

Serum levels of HA, COMP, and MMP-3 were evaluated at baseline and 3 months after injection. The amount of HA decreased significantly after 3 months (Fig. 8A), which indicates a notable decline in cartilage degeneration (*p* < 0.05). The levels of COMP were reduced after the injection of AD-MSCs (Fig. 8B). Cartilage oligomeric matrix protein (COMP) concentration significantly decreased in patients who underwent the AD-MSCs injection compared with the control group (*P* < 0.05). No significant changes were observed in MMP-3 levels compared to the control group after AD-MSC injection (*P* = 0.104) (Fig. 8C).

**Fig. 9:**
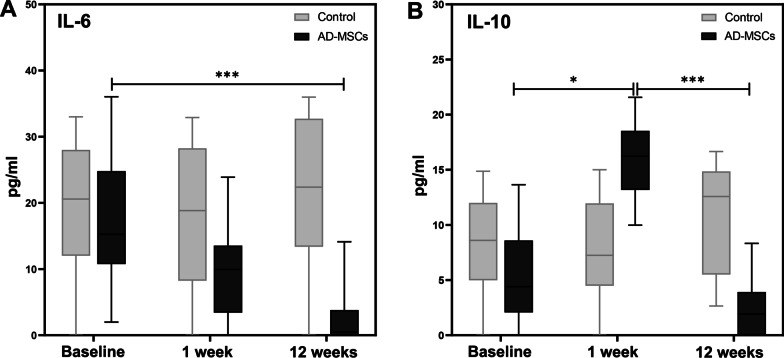
Determination of inflammatory biomarkers in blood serum. **A** The level of IL-6 reduced significantly after 12 weeks of the injection in AD-MSCs group (*P* < 0.001). **B** IL-10 increased during the first week of injection (*P* < 0.05) and then decreased significantly 12 weeks after injection (*P* < 0.001). Data markers represent mean values; error bars, 95% confidence interval; *P < 0.05, ***P < 0.001, between and within groups; and one-way repeated measures analysis of variance (ANOVA)

Detection of inflammatory cytokines (IL-6 and IL-10) before and 1 week and 3 months after injection is shown in Fig. 9A, B, respectively. The expression level of IL-6 in the AD-MSCs group decreased dramatically after 3 months and was significant compared to the control group (*P* < 0.001). Illustrated in Fig. 9B, a significant increase in the levels of IL-10 were seen in the AD-MSCs group 1 week after injection (*P* < 0.05). After 3 months, a decreasing trend could be seen in the expression of this biomarker, which is significantly compared to the control group (*P* < 0.001).

### Cell surface CD markers assessment in blood samples

**Fig. 10:**
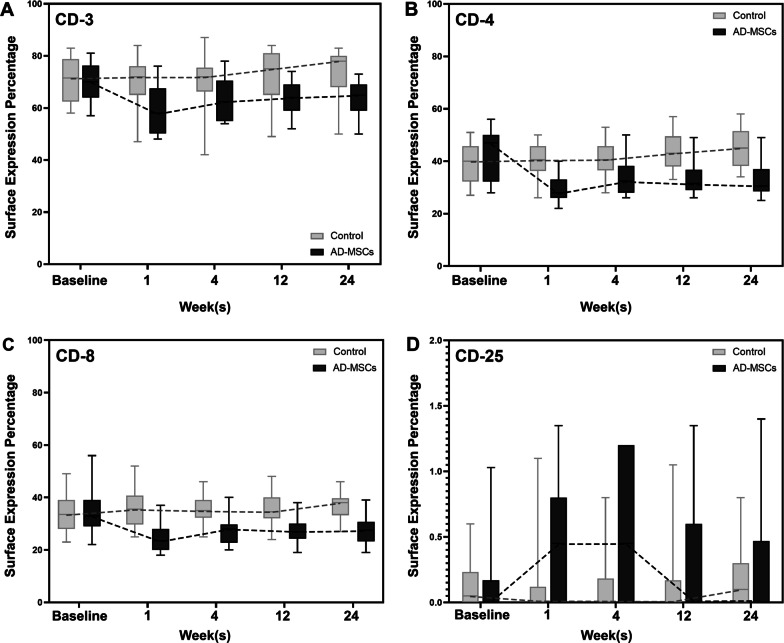
Cell surface CD marker expression during the 6-month follow-up. **A** The percentage of cells expressing CD3 decreased significantly in AD-MSCs group (*P* < 0.005). Surface expression percentages of **B** CD4 and **C** CD8 followed a noticeable decreasing trend during the 6-month follow-up in AD-MSCs group (*P* < 0.001). **D** CD25 expression increased dramatically in AD-MSCs group in comparison with the control group (*P* < 0.005). Data markers represent means; error bars, 95% confidence interval; and statistical analysis conducted by the one-way repeated measures analysis of variance (ANOVA)

The evaluation of CD3, CD4, and CD8 markers in the AD-MSCs group at different time points had almost the same trend. The levels of cell surface markers in the blood samples of patients receiving AD-MSCs during the 6-month follow-up had a significant reduction (Fig. 10). This decreasing trend in all three markers was significant, and the *P* value for CD3, CD4, and CD8 changes in the AD-MSCs group compared to the control group is equal to *P* < 0.005, *P* < 0.001, and *P* < 0.001, respectively. Also, the amount of CD25 in the AD-MSCs group increased, remarkably (*P* < 0.005).

## Discussion

The present study investigated the safety and efficacy of intra-articular injection of allogeneic AD-MSCs in KOA by the assessment of clinical outcomes, MRI, some serum biomarkers, and cell CD markers in peripheral blood samples. Clinical outcome measures showed improvement in AD-MSCs group.

Knee osteoarthritis is the most prevalent type of OA which has a distinct effect on the global health status [34]. Despite the availability of different conventional therapeutic methods for KOA, generally classified as surgical and non-surgical treatments, finding an innovative treatment with regenerative ability and inflammation suppression has remained non accessible. Intra-articular transplantation of AD-MSCs has been proposed as a novel minimally invasive treatment for KOA by inducing cartilage regeneration beside inflammation suppression [35, 36]. A high proliferation rate (tolerance for multiple passages without losing their stemness properties), abundance and accessibility (can be obtained through a minimally invasive liposuction procedure), strong immunomodulatory properties (low immunogenicity and secretion of anti-inflammatory cytokines and growth factors), and chondrogenic potential have made AD-MSCs a promising option for KOA [37, 38]. The other sources of MSCs may also be effective; for instance, bone marrow is the most studied source of MSCs which has shown promises in KOA, but the superiority of AD-MSCs in comparison with BMSCs has been reported in the recent studies [18]. Umbilical cord-derived mesenchymal stromal cells (UC-MSCs) are the other one which it is potential in KOA examined in some studies which indicated the positive results with high proliferation rate and low risk of viral contamination [30, 39]. However, UC-MSCs have been introduced inferior to AD-MSCs in a meta-analysis which compared the efficacy of MSCs with different sources in KOA [40]. Moreover, synovial fluid and dental pulp are the other uncommon sources of MSCs due to the lack of sufficient number of MSCs and invasive harvesting procedure [41].

WOMAC, KOOS, VAS, and SF-36 are the most common questionnaires available to evaluate the effectiveness of treatment modalities in patients with KOA [42]. These questionnaires were used in our study to evaluate the relevant changes in the patients’ health conditions during the 12-month follow-up. Assessment of these questionnaires together indicates the improvement of the patients’ condition in AD-MSCs group.

WOMAC is one of the most common questionnaires used in similar studies [18]. The results of the present study have shown that the WOMAC score in the AD-MSCs group represented a remarkable decrease during 12 months of follow-up compared to the control group, and the mean score of this questionnaire in the AD-MSCs group 6 months after injection decreased by more than 70%. Despite the temporary decrease in WOMAC score in the control group after 3 months, this value increased remarkably and reached the baseline score. In this context, Lee and colleagues used this questionnaire at baseline and 3, and 6 months after intra-articular injection in order to investigate the effectiveness of autologous AD-MSCs in KOA. Results of their study showed remarkable decline in WOMAC score and pain reduction 6 months after injection [26].

In KOOS, contrary to WOMAC, the lower score describes the worse health status of the patient. The results of our study showed a noticeable increase in the scores of all subgroups (symptoms, pain, daily function, sport and recreation, and quality of life), in the AD-MSCs injected group during the 1-year follow-up. This increasing trend was significant, while in the control group, despite a slight increase after 3 months, at the end of 12 months, no significant changes were observed compared with the baseline.

According to the VAS results, the severity of pain indicated by the patients in AD-MSCs group during the 1-year follow-up showed a decreasing trend, and this reduction was noticeable. A meta-analysis of nine randomized controlled trials with 476 patients evaluated pain level and functionality improvement after intra-articular injection of MSCs in KOA. The results of this study indicated that transplantation of MSCs significantly reduced the VAS score during the long-term follow-up [43].

According to the assessment of SF-36, the condition of the patients who received AD-MSCs was improved significantly, and this improvement was remarkable in all subgroups of the SF-36.

Assessment of the questionnaire-based outcomes indicated almost the same trend during the 12-month follow-up in similar studies. The highest rate of improvement was observed 3 months post-injection then plateaued between 6 and 12 months after AD-MSCs injection. The observed trend in this study was the same as the other studies. For example, the WOMAC and KOOS outcomes in the study conducted by Freitag et al. followed a similar trend. They used single and double doses of 100 × 10^6^ AD-MSCs in the patients with KOA. The WOMAC score and all subgroups of KOOS in the single dose group followed an improving trend up to month 3 after injection and then remained stable up to 12 months [29]. Furthermore, the results of VAS and WOMAC in the other study conducted in 2021 indicated the same pattern. Three doses (16 × 10^6^, 32 × 10^6^, and 64 × 10^6^) of AD-MSCs administrated in this trial. The WOMAC total score and VAS in all intervention groups increased dramatically 3 months after injection, while reached an approximately constant value at 6, 9, and 12 months [44]. The paracrine effect of injected MSCs is certainly one potential mechanism that could be contributing to the observed outcomes. Remarkable increase in the clinical outcomes 3 months after injection was observed, probably due to the paracrine ability of MSCs by secretion of immunomodulatory cytokines and growth factors [45].

MRI findings demonstrated thickness alteration in 12 different points of the femoral and tibial articular cartilage. These 12 points of articular cartilage were selected according to the cartilage areas assessed in whole-organ magnetic resonance imaging score (WORMS) in KOA [46]. Evaluation of the results of these changes indicated a significant increase in the cartilage thickness in the AD-MSCs group in just two points of the tibia (TMP and TMA). Nevertheless, in the other points, despite the increasing trend of cartilage thickness in the AD-MSCs group, any statistically meaningful different was not observed between the AD-MSCs and control groups. Considering the increasing trend of cartilage thickness in the AD-MSCs group in most of the mentioned points (except for the antero-lateral femur and tibia articular cartilage, where the thickness decreased after 1 year), future studies with large population are suggested to show the possible improvement. In a similar clinical trial, Khalifeh Soltani et al. evaluated the chondral thickness by the use of magnetic resonance arthrography (MRA) after the 6 months of allogeneic placental MSCs transplantation in KOA. Their results declared nearby 10% increase in chondral cartilage thickness after 6 months in case group [47].

Also, articular cartilage evaluation by MRI has considered in the similar randomized trials by the use of common scoring systems. For instance, in 2019, after intra-articular injection of autologous AD-MSCs, researchers examined the modification of articular cartilage based on MRI osteoarthritis knee scores (MOAKS) between two treatment groups (a single dose of 100 × 10^6^ AD-MSCs and double doses of 100 × 10^6^ with 6-month intervals), and control group. Their results showed that 12 months after the first injection, the symptoms were alleviated in the patients receiving double doses of AD-MSCs, while in spite of the improvement in the MOAKS, it was not significant [29]. Furthermore, the results of the study conducted by Zhao et al*.* with 18 participants in three different doses, who underwent intra-articular injection of allogeneic human AD-MSCs (the low-dose, mid-dose, and high-dose received groups with 1.0 × 10^7^, 2.0 × 10^7^, and 5.0 × 10^7^ cells, respectively), indicated that significant improvement in the symptoms was evaluated by WOMAC and SF-36, and structural modifications of articular cartilage were confirmed by multi-compositional MRI sequences. However, any remarkable difference has not been reported in WORMS between groups [19]. However, none of the mentioned studies reported a separate score of the changes in cartilage thickness. In this regard, considering cartilage thickness and volume in future studies for more accurate monitoring of the MSCs effects in cartilage regeneration in KOA is proposed.

Assessment of biological markers is indicated for the early diagnosis of inflammatory diseases such as OA, and also for monitoring the potency of the treatment methods [48]. In the present study, three OA specific biomarkers (COMP, HA, and MMP-3) and two inflammatory cytokines (IL-10 and IL-6) in blood samples were investigated in different time points. Noticeable reduction of COMP level in the blood serum of the patients in AD-MSCs group after 3 months indicated cartilage damage alleviation and the effectiveness of the transplantation of AD-MSCs in KOA. Yang et al*.* [49] in a OA cohort study showed that COMP is a promising predictor of cartilage destruction, which its higher level usually associates with synovitis in KOA. Furthermore, An et al. in a preclinical study indicated that human AD-MSCs administration leads to the reduction of elevated COMP serum concentration in KOA rabbits [50].

In addition, the expression level of HA in AD-MSCs group diminished significantly and the average serum level of HA among the patients who received AD-MSCs injection, decreased by 47.18% after 3 months compared with the baseline. The correlation between serum HA concentration and OA has been evidenced in numerous literatures [51, 52]. Serum HA has been proposed as an alternative tool besides conventional diagnostic methods for early detection of KOA and monitoring the disease progression [53].

Matrix metalloproteinase-3 (MMP-3) is another early KOA biomarker investigated in this study. Despite a slight decrease in the concentration of MMP-3 in the AD-MSCs group after 3 months, any significant difference was not reported between AD-MSCs and control group. The results of some studies conducted for the assessment of any association between MMP-3 level and OA severity indicated that the elevated level of MMP-3 correlates with severe OA [54–56]. Nonetheless, MMP-3 is an enzyme that is involved in the degradation of various ECM proteins. Its association has been reported in other physiological and pathological conditions and is not specific to cartilage degradation [57]. However, numerous preclinical studies investigated serum level of MMP-3 in osteoarthritis after MSCs transplantation in animal models. For instance, Lee et al. [58] evaluated the level of MMP-3 after using membrane-free components of AD-MSCs in OA rats. They proposed that reducing trend of MMP-3 levels may be an indicator for alleviation of cartilage destruction after applying this treatment method. Furthermore, the reduction of MMP-3 expressed by chondrocytes was reported in another animal study after BMSCs transplantation in New Zealand rabbits in KOA model [59]. In spite of the MMP-3-level assessment following the MSCs transplantation in KOA in different animal models, to the best of our knowledge no clinical trial evaluated the changes of this marker after MSCs therapy in KOA. In the present study, in spite of the significant reduction in serum COMP and HA, any meaningful changes in MMP-3 serum concentration were not observed. Hyaluronic acid (HA) is the main component of the cartilage ECM, and COMP is a protein that is produced by chondrocytes and is specifically presented in cartilage ECM, while MMP-3 is not specific to cartilage degradation [57, 60]. Hence, the serum levels of COMP and HA may be more correlated with OA progress or improvement compared to the MMP-3 serum concentration. Although no significant changes in MMP-3 level were reported, a slight decreasing trend in the AD-MSCs group may suggest evaluation of MMP-3 serum concentration in future studies with larger population.

The serum level of IL-6, an upstream pro-inflammatory cytokine with complex roles in diseases with inflammatory background, was declined after MSCs transplantation in consequence of modulating immune system [61]. Serum concentration of IL-6 is proposed as an indicator of OA severity, and its association with cartilage destruction is endorsed in many clinical investigations [62]. In this regard, Li and colleagues evaluated the level of this marker as a post-treatment indicator after the injection of BMSCs in patients with KOA. They showed that the secretion of this marker significantly declined at 6 and 12 months after injection [22]. This inflammatory marker has also been examined in the present study, and the results showed that the expression level of IL-6 in the AD-MSCs group significantly decreased during the 3-month follow-up, indicating that AD-MSCs by immune system modulation alleviated the inflammation triggered by KOA.

Interleukin-10 is the other inflammatory cytokine which MSC therapy affects its secretion. The IL-10 expression increased by MSCs in order to reduce the proliferation of T cells to modulate the immune system. This anti-inflammatory cytokine along with other markers such as TGF-β1 plays a crucial role in the regulation of immune system by inhibition of lymphocytes proliferation and maturation of dendritic cells [63]. In this respect, Sofia et al*.* [64] have shown that serum IL-10 levels were increased in OA rats after treatment with Wharton jelly-derived mesenchymal stromal cells (WJ-MSCs). Regarding this anti-inflammatory cytokine, our results showed that in the AD-MSCs group, a significant elevation in IL-10 expression was observed after 1 week, which indicates the impact of AD-MSCs in down-regulation of the immune system to avoid rejection after allogeneic AD-MSCs transplantation. Afterward, expression of this cytokine showed a remarkable decline after 3 months, which may be occurred due to reduction of pro-inflammatory cytokine (IL-6). The pro-inflammatory and anti-inflammatory cytokines are in a complex interaction. According to the previous basic studies, IL-6 has been shown to stimulate the production of IL-10 to regulate the immune response and prevent excessive inflammation. Hence, IL-6 reduction leads to the decrease in signal transducer and activator of transcription 3 (STAT3) activation rate (the most common pathway that has been implicated in the promotion of IL-10 secretion) which results in the inhibition of IL-10 expression [65, 66]. In the control group, no changes in the secretion of IL-10 were reported.

To show that if AD-MSCs can lead to the reduction of inflammation in KOA, the percentage of cells expressing CD3, CD4, CD8 and CD25 was analyzed in blood. These CD markers may be suggested as additional inflammatory indicators in OA, due to the recent reports that elucidate T helper (Th) cells which are involved in the pathogenesis of OA [67]. Synovial fluid analysis could reflect the changes in inflammation status better, but collecting the peripheral blood sample is safe, more feasible, and noninvasive than synovial fluid samples. Moreover, the findings of several studies indicated correlation between synovial fluid and peripheral blood CD markers in patients with OA [68, 69].

In our study, the results of the evaluation of CD3, CD4, and CD8 in the blood serum of patients who received AD-MSCs had almost followed a similar decreasing trend at different time points during the 6-month follow-up. A dramatic reduction in the percentages of cells expressing these markers accrued after 1 week due to the immune-regulatory features of AD-MSCs. Then, approximately a plateau has been observed in the trend of these markers during 6-month follow-up. The expression level of each of these markers reached a relatively stable values, which were well below the baseline levels between 3 and 6 months after injection. This decrease in the expression of the mentioned cell surface markers 6 months post-injection in the AD-MSCs group, compared with the baseline levels, can be attributed to the alleviation of KOA-induced inflammation. While in the control group, the levels of CD3 and CD8 were almost constant during the 6 months, the percentage of CD4^+^ cells increased slightly 3 months after injection, which are in association with other findings investigated in this study such as the results of OA index biomarkers and various analyzed questionnaires. Enhancement in CD4^+^ cells population is possibly caused by the progression of OA. Furthermore, CD25^+^ cells increased dramatically in the AD-MSCs group in comparison with the control group. CD25 is usually expressed in low levels in humans and is one of the markers expressed by T regulatory (Treg) cells. Treg cells are a subgroup of T cells that modulate immune system responses by inhibiting the proliferation of T cells and the secretion of anti-inflammatory cytokines such as IL-10 and TGF-β. It has been shown that MSCs contribute to the activation of Treg cells in order to modulate the immune system [70].

The results of our study showed that single intra-articular injection of 100 × 10^6^ allogeneic AD-MSCs was not associated with any SAEs which is consistent with the results of studies which used the same cell dosage [26–28]. Additionally, considering the inflammatory markers assessment besides the wide range of outcome measures revealed the remarkable efficacy of this method in KOA. The improvement in clinical outcomes such as WOMAC, VAS, KOOS, and SF-36 was reported by most of the similar clinical trials which evaluated autologous and allogeneic MSCs [20, 71]. However, the expression of two inflammatory serum markers following MSCs therapy was reported only by one study, Li et al*.* in 2020, which evaluated the inflammation modulation after MSCs administration in KOA. Their finding revealed the remarkable reduction in TNF-α and IL-6 levels in MSCs group indicating decrease in the inflammation which was in accordance with our results [22].

## Conclusion

Translation of this multiple assessment findings proposed that intra-articular transplantation of AD-MSCs alleviates KOA complications due to inflammation modulation and induction of cartilage regeneration. Administration of allogeneic AD-MSCs is safe and improves clinical signs and symptoms. The analyzed end points suggested pinnacle enhancement in WOMAC, VAS, KOOS, and SF-36 after 6 months. For future investigations, clinical trials with large populations, considering repeated booster doses with 6-month intervals, and assessment of more biological markers in synovial fluid following the AD-MSCs transplantation are recommended.

## Data Availability

All data generated or analyzed during this study are included in this published article.

## Abbreviations

AD-MSCs: Adipose tissue-derived mesenchymal stromal cells
AEs: Adverse events
AP: Anterior–posterior
BMI: Body mass index
BMSCs: Bone marrow-derived mesenchymal stromal cells
CBC: Complete blood count
CRP: C-reactive protein
COMP: Cartilage oligomeric matrix protein
ELISA: Enzyme-linked immunosorbent assay
ESR: Erythrocyte sedimentation rate
FSPGR: Fast spoiled gradient-echo
FLC: Femur lateral central
FLP: Femur lateral posterior
FLA: Femur lateral anterior
FMC: Femur medial central
FMP: Femur medial posterior
FMA: Femur medial anterior
GCP: Good clinical practice
GMP: Good manufacturing practice
HA: Hyaluronic acid
ICC: Intraclass correlation coefficient
IL-6: Interleukin-6
IL-10: Interleukin-10
IRCT: Iranian registry of clinical trials
IL1β: Interleukin 1β
IR-FDA: Iran food and drug administration
KOA: Knee osteoarthritis
KOOS: Knee osteoarthritis outcome score
K-L: Kellgren–Lawrence
MSCs: Mesenchymal stromal cells
MHC-II: Major histocompatibility complex class II
MHC-I: Major histocompatibility complex class I
MMP-3: Matrix metalloproteinase-3
MRI: Magnetic resonance imaging
MOAKS: MRI osteoarthritis knee scores
MANOVA: Multivariate analysis of variance
MRA: Magnetic resonance arthrography
NS: Normal saline
NOS: Not otherwise specified
OA: Osteoarthritis
PBS: Phosphate buffer saline
PRP: Platelet-rich plasma
SF-36: Short Form 36
SAE: Serious adverse events
STAT3: Transducer and activator of transcription 3
UC-MSCs: Umbilical cord-derived mesenchymal stromal cells
TLA: Tibia lateral anterior
TLC: Tibia lateral central
TLP: Tibia lateral posterior
TMA: Tibia medial anterior
TMC: Tibia medial central
TMP: Tibia medial posterior
Th: T helper
Treg: T regulatory
TGF-β1: Transforming growth factor β1
TNF-α: Tumor necrosis factor-α
VAS: Visual analogue scale
WOMAC: Western Ontario and McMaster Universities Osteoarthritis Index
WORMS: Whole-organ magnetic resonance imaging score
WJ-MSCs: Wharton jelly-derived mesenchymal stromal cells
